# General self-efficacy as a moderator between severity of menopausal symptoms and satisfaction with life in menopausal women

**DOI:** 10.3389/fpubh.2024.1426191

**Published:** 2024-08-29

**Authors:** Agnieszka Bień, Iwona Niewiadomska, Magdalena Korżyńska-Piętas, Ewa Rzońca, Marta Zarajczyk, Beata Pięta, Krzysztof Jurek

**Affiliations:** ^1^Chair of Obstetrics Development, Faculty of Health Sciences, Medical University of Lublin, Lublin, Poland; ^2^Department of Social Psychoprevention, John Paul II Catholic University of Lublin, Lublin, Poland; ^3^Faculty of Health Sciences, Department of Obstetrics and Gynecology Didactics, Medical University of Warsaw, Warsaw, Poland; ^4^Department of Mother and Child Health, Poznań University of Medical Sciences, Poznań, Poland; ^5^Institute of Sociological Sciences, John Paul II Catholic University of Lublin, Lublin, Poland

**Keywords:** menopause, women, self-efficacy, satisfaction with life, quality of life

## Abstract

The study aimed to determine whether self-efficacy acts as a moderator between the severity of menopausal symptoms and life satisfaction. The research tools were: The Generalized Self-Efficacy Scale (GSES), The Satisfaction with Life Scale (SWLS) The Menopause – Specific Quality of Life (MENQOL), and a standardized questionnaire comprising questions on the participants’ characteristics. 516 women using health care services who had menopausal symptoms took part in the study. Self-efficacy (GSES) (*r* = −0.176; *p* < 0.001) and life satisfaction (SWLS) (*r* = −0.106; *p* = 0.016) were negatively correlated with the severity of menopausal symptoms (MENQOL). The self-efficacy correlated positively with satisfaction with life (*r* = 0.278; *p* < 0.001). A low level of self-efficacy was associated with a significant and negative relationship between the severity of menopausal symptoms and satisfaction with life (*p* = 0.005), while at a high and moderate level of self-efficacy, the severity of menopausal symptoms was not associated with life satisfaction (*p* > 0.05). Self-efficacy moderates the relationship between the severity of menopausal symptoms and life satisfaction in menopausal women. The moderating function of self-efficacy is that, at its low level, an increase in the severity of menopausal symptoms reduces life satisfaction, while at medium and high levels, an increase in the severity of menopausal symptoms does not reduce life satisfaction.

## 1 Introduction

Menopause is the term used to describe the natural, permanent end of menstruation as a result of the cessation of ovarian function. The range of symptoms that appear, their severity, and the timing of menopause is highly individual (1). In women, it mostly occurs between the ages of 45 and 55. The gradual extinction of the hormonal function of ovaries leads to numerous ailments and problems in woman’s physical, psychological, and social domains of life (1, 2). Physical symptoms include hot flashes, night sweats, palpitations, weight gain, sleep disorders, and physical exhaustion. Mental problems include anxiety, fear, depression, sadness, emotional lability, poor attention span, feeling lack of sense of life. Symptoms in social functioning, among others, include problems in intimate relationships, and difficulties in fulfilling social roles (1–5).

A number of the indicated symptoms can significantly affect the functioning and well-being of menopausal women. As a result, quality of life and perceptions of life satisfaction change among women during this period of life (6–8). Quality of life is a complex concept with many definitions depending on the discipline of interest. It includes assessing perceptions of life using objective indicators such as income, education level, living conditions, and access to medical care (9–11). In turn, health-related quality of life focuses on the consequences, and implications of quality of life resulting from health status, as well as holistic well-being (12). The Menopause-Specific Quality of Life Questionnaire (MenQoL) is a questionnaire that assesses the quality of life of menopausal women, taking into account the specificity and severity of symptoms and experiences related to this period (13). Life satisfaction is a concept resulting from an individual’s comparison of his/her current life situation with his/her accepted standards based on subjective feelings and life experiences. In peri-menopausal women, the level of sense of satisfaction with life may change due to emerging changes in the health situation, limitation of physical activity resulting from somatic symptoms typical for this period of life. Therefore, the level of satisfaction with life becomes very important for the global assessment of a individual’s quality of life. In conclusion, quality of life is more external and measurable through specific indicators, while satisfaction with life is an individual’s internal, subjective feeling about his or her own life. These two concepts are related, but not defined at the same level - it is possible to have high quality of life and declared low satisfaction with life, and vice versa. Although the two concepts are related, they can also function as two separately assessed characteristics (14–17). In the search for factors that contribute to maintaining high life satisfaction in menopausal women, it is useful to highlight the importance of the psychological factor in the form of self-efficacy (6–8). Generalized self-efficacy is a belief in one’s ability to mobilize cognitive resources, competencies, learned solutions and effort needed to effectively perform a task/take action in various types of stressful circumstances (18, 19). People with a higher level of self-efficacy are more likely to treat adversities as challenges, are more optimistic, motivated to take action, involved in problem solving, controlling the course of events and feel satisfied with the tasks they complete. All of these characteristics contribute to an improved quality of life (8, 20, 21).

Women in the menopausal period may experience a variety of emotions, including anxiety about the physical and/or psychological menopausal symptoms, helplessness due to changes in family and career functioning deprivation of important biological and/or psychological needs. This may be influenced by factors such as the sandwich generation, empty nest syndrome, and prevalence of anxiety (22, 23). The problems may co-occur or overlap at a particular time or over a long period, resulting in a number of negative emotions. The above stressors, negatively influencing women’s feelings, can exacerbate the already occurring unfavorable symptoms of menopause for a woman’s psyche (24, 25).

The aim of the study was to assess the self-efficacy and satisfaction with life of women during menopause and to determine whether self-efficacy acts as a moderator between the severity of menopausal symptoms and life satisfaction.

The authors proposed two hypotheses in an attempt to answer the question of whether women’s responses to menopausal symptoms may be optimized.

1. Decreased life satisfaction in menopausal women is significantly associated with an increase in menopausal symptoms.
2. Self-efficacy has a moderating function in menopausal women for the relationship between the severity of menopausal symptoms and life satisfaction. Increased levels of self-efficacy act as a protective factor against the negative effect of menopausal symptoms on life satisfaction.

## 2 Materials and methods

The research was conducted from April 2020 to March 2021. The study included 516 female patients who were using health care services in the Lubelskie Voivodeship (Poland). The inclusion criteria for the study were as follows: women experiencing menopausal symptoms, aged between 41 and 60, not having used any menopause-related treatments (such as hormone therapy, selective estrogen receptor modulators, aromatase inhibitors, and soybean extracts) in the 3 months prior to the study, and speaking Polish as their first language. Induced or surgical menopause (hysterectomy, ovarian excision, radiotherapy, and chemotherapy), the presence of chronic disorders such as kidney impairment, immunodeficiency, cardiovascular disease, mental illness, and untreated thyroid disease were all excluded criteria for the study.

Menopausal status was defined in accordance with the *World Health Organization’s* (WHO’s) classification (26). To elucidate this distribution, women with regular menstrual bleeding during the last year were classified as premenopause, those with irregular bleeding during the last 12 months as perimenopause, and those with amenorrhea during the last year as menopause. Finally, women were classified as post-menopause, if they had no menstrual bleeding from 1 year and above (26). 516 correctly completed questionnaires out of the 540 distributed were further analyzed and the success rate of the data obtained was 95.55%.

Participants completed a set of questionnaires and then return them personally to research assistants. A questionnaire technique and a diagnostic survey method were used in the study. The research tools were: The Generalized Self-Efficacy Scale (GSES), The Satisfaction with Life Scale (SWLS), The Menopause – Specific Quality of Life (MENQOL), and a standardized interview questionnaire comprising questions on the participants’ characteristics. The study was performed in accordance with the Helsinki Declaration. The study protocol and ethics of this study were approved by the Lublin Medical University Bioethics Committee (approval no. KE-0254/257/2020), which was based on Guidelines for Good Clinical Practice. Respondents were informed about the aim of the study, voluntary participation, and that study results were anonymous and to be used exclusively for research purposes.

**Generalized Self-Efficacy Scale** – developed by Schwarzer and Jerusalem, adapted to Polish conditions by Juczyński. This tool is used to measure the belief in one’s ability to deal effectively with difficult situations and to cope with adversity. The total score of the GSES scale is the sum of all points obtained from 10 statements to be responded on a scale of 1 to 4. The respondents received ratings ranging from 10 to 40 points. The raw score is transformed into standardized sten norms, with low scores being 1–4 sten (10–24 points), average scores being 5–6 sten (25–29 points), and high scores being 7–10 sten (30–40 points). Cronbach’s alpha for scale reliability is 0.85 (27).

**The Satisfaction with Life Scale** by Diener, Emmons, Larson and Griffin, adapted to Polish conditions by Juczyński. It is used to measure satisfaction with life, both by healthy and sick people, by comparing the conditions of their lives with self-imposed standards. The scale consists of 5 statements rated on a seven-point scale. A number of 1 indicates that the respondent entirely disagrees with the supplied statement, and a value of 7 indicates that they completely agree. The total score, which ranges from 5 to 35 points, is the aggregate of all ratings. The respondent’s level of life satisfaction increases as the score rises. The questionnaire is 0.82 percent reliable as determined by Cronbach’s alpha (28).

**The Menopause-Specific Quality of Life Questionnaire** developed by Hilditch et al., has 29 sub-items and a 7-point scale ranging from 0 to 6. Each sub-item comprises four domains: vasomotor (items 1–3), psychosocial (items 4–10), physical (items 11–26), and sexual (items 27–29), and each sub-item includes a symptom that may arise during menopause. Respondents indicate whether or not a specific issue has affected them in the previous month. If yes, they state how much this issue upset them on a scale of 0 to 6, with 0 denoting that it bothered them not at all and 6 denoting that it bothered them greatly. With increasing MENQOL scores, levels of bother experienced from the symptom are increased as well. The reliability of the questionnaire is 0.8 (13).

### 2.1 Statistical methods

**Figure 1:**
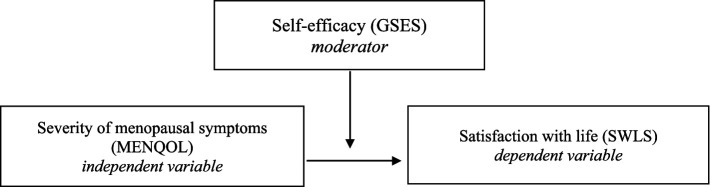
Conceptual diagram of a moderation model in which the effect of the severity of menopausal symptoms (MENQOL) on the satisfaction with life (SWLS) is dependent on a self-efficacy (GSES).

Categorical variables were presented as percentages (%) and numbers (n). Quantitative variables with normal distribution were presented as mean (M) and standard deviations (SD). A descriptive analysis of the variables of the study was performed. The Pearson correlation coefficient (Pearson’s r) was used to evaluate the relationship between the studied variables. A bias-corrected bootstrap estimation (10,000 samples) with a confidence interval of 95% was used to evaluate the moderating role of self-efficacy (GSES) between the severity of menopausal symptoms (MENQOL) and satisfaction with life (SWLS) (Figure 1). For the analysis, SPSS 28 statistic software and PROCESS (model 1) for SPSS was used. The Johnson–Neyman technique was used to probe it. The technique seeks to find the value or values of the moderator (W) within the data, if they exist, such that the *p*-value for the ratio of the conditional effect of the focal predictor at that value or values of W is exactly equal to 0.5.

## 3 Results

The study included 516 women aged between 41 and 60 years. The mean age was 50.24 years (SD = 3.96). The majority of women lived in urban areas (68.6%), were married (81.4%), had a higher education qualification (64.0%), were working (81.4%), and described their financial situation as average (88.2%) – Table 1.

**Table 1:** Participant demographics.

Characteristics		*n*	%
Mean age / years (M, SD)		50.24 (3.96)	
Place of residence	Urban area	345	68.6
	Rural area	162	31.4
Marital status	Single/divorced/widowed	96	18.6
	Married	420	81.4
Education	Primary	40	7.8
	Secondary	146	28.3
	Higher	330	64.0
Professional status	Working	420	81.4
	Not working	96	18.6
Financial situation	Bad, rather bad	52	10.1
	Average	455	88.2
	Rather good, good	9	1.7

M, mean; SD, standard deviation.

In women mean self-efficacy (GSES) was 29.44 ± 4.20, satisfaction with life (SWLS) was 20.93 ± 5.45 and overall quality of life (MENQOL) was 4.16 ± 1.47. The severity of menopausal symptoms (global score MENQOL) had a negative correlation with self-efficacy (GSES) (*p* = 0.001) and satisfaction with life (SWLS) (*p* = 0.016). There was a negative correlation between the vasomotor, psychosocial, and sexual domains of respondents’ quality of life and their sense of self-efficacy, and a negative correlation between the psychosocial and sexual domains of respondents’ quality of life and their life satisfaction. The correlations took values from −0.244 to −0.082. A positive correlation was also found between self-efficacy and satisfaction with life (*p* < 0.001) – Table 2.

**Table 2:** Correlation between overall quality of life, self-efficacy and life satisfaction of respondents.

		M (SD)	GSES		SWLS	
			*r*	*p*	*r*	*p*
GSES		29.44 (4.20)	**–**	**–**	**–**	**–**
SWLS		20.93 (5.45)	0.278	p < 0.001	**–**	**–**
MENQOL	Global score	4.16 (1.47)	−0.176	0.001	−0.106	0.016
	Vasomotor	3.69 (2.09)	−0.089	0.044	−0.082	0.064
	Psychosocial	4.67 (1.71)	−0.244	0.000	−0.152	0.001
	Physical	3.95 (1.68)	−0.070	0.113	0.019	0.668
	Sexual	4.33 (2.26)	−0.140	0.001	−0.101	0.022

GSES, generalized self-efficacy scale; SWLS, satisfaction with life; MENQOL, the menopause specific quality of life questionnaire.

Then, the moderating role of self-efficacy between the severity of menopausal symptoms and satisfaction with life was evaluated. The severity of menopausal symptoms was negatively and significantly associated with life satisfaction (*p* = 0.005). A positive correlation was also found between the severity of menopausal symptoms and self-efficacy (*p* = 0.009), while the relationship between self-efficacy and life satisfaction was negative but insignificant (*p* > 0.05) – Table 3.

**Table 3:** The self-efficacy (GSES) as a moderator of the relationship between the severity of menopausal symptoms (MENQOL) and satisfaction with life (SWLS) — model summary.

	Unstandardized coefficient	SE	*T*	*p*	LLCI	ULCI
Intercept	23.460	4.902	4.786	<0.001	13.829	33.091
MENQOL	−2.886	1.034	−2.792	0.005	−4.917	−0.855
GSES	−0.046	0.160	−0.286	0.775	−0.361	0.269
MENQOL x GSES	0.089	0.034	2.610	0.009	0.022	0.156
*R* = 0.304; Model *R*^2^ = 0.093; F (3;511) = 17.381; *p* < 0.001; MSE = 27.07						

CI, confidence interval; LL, lower limit; UL, upper limit.

**Figure 2:**
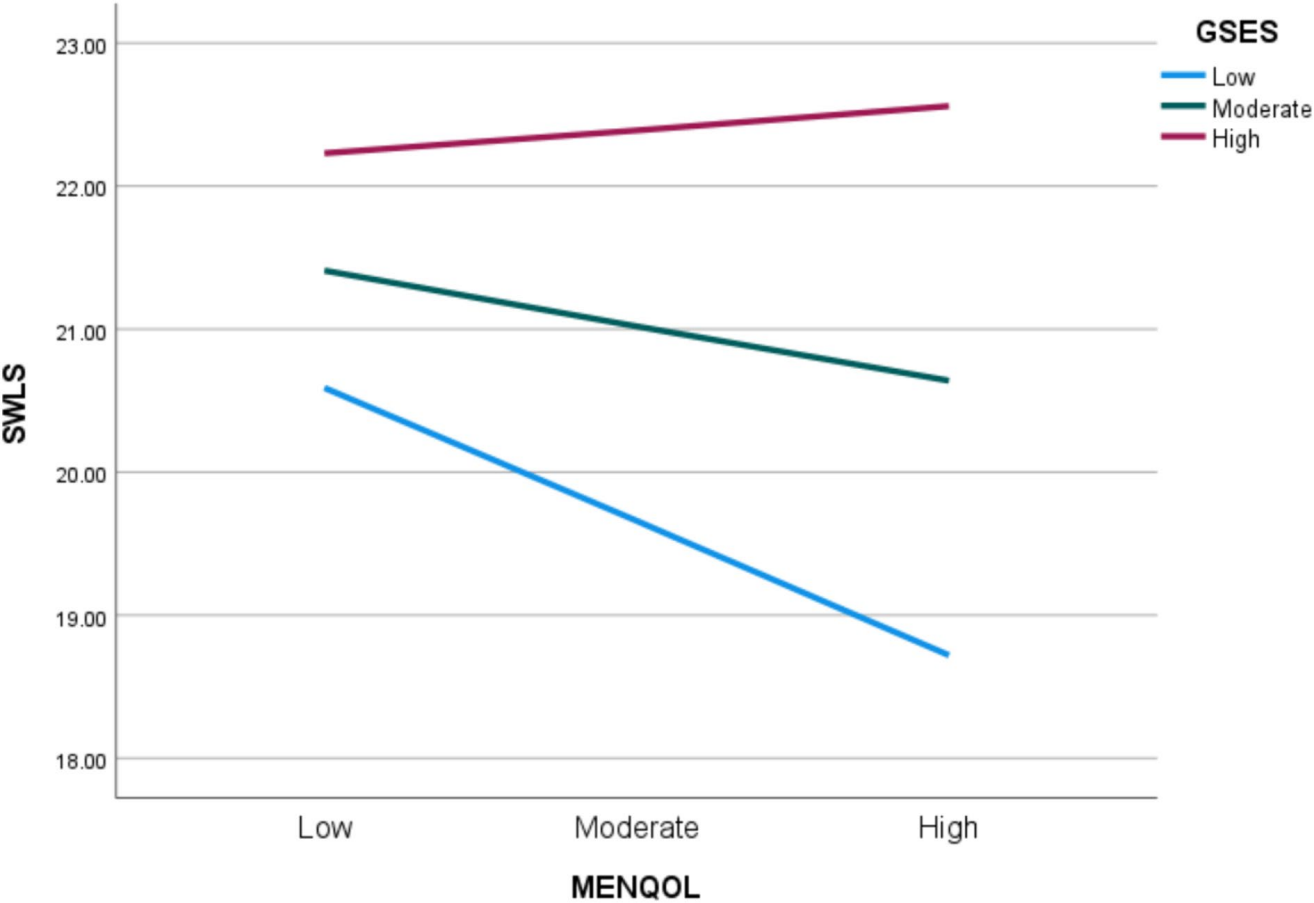
Satisfaction with life (SWLS) explained by the interaction between the severity of menopausal symptoms (MENQOL) and self-efficacy (GSES).

Regarding the moderation effect, a low level of self-efficacy is associated with a significant and negative relationship between the severity of menopausal symptoms and satisfaction with life (*p* = 0.005), while at high and moderate level of self-efficacy, the severity of menopausal symptoms is not associated with life satisfaction (*p* > 0.05) (Figure 2; Table 4). The self-efficacy explains 1.2% of the variance in the interaction.

**Table 4:** Conditional effects of the severity of menopausal symptoms in the satisfaction with life for specific values of self-efficacy.

	GSES	Effect	SE	T	P	LLCI	ULCI
MENQOL	- 1 SD	−0.636	0.225	−2.827	0.005	−1.078	−0.194
	*M*	−0.261	0.160	−1.637	0.102	−0.575	0.052
	+ 1 SD	0.113	0.204	0.556	0.579	−0.287	0.514

CI, confidence interval; LL, lower limit; UL, upper limit.

The relationship between self-efficacy and satisfaction with life is significantly influenced by the level of self-efficacy within a certain range. Self-efficacy values ranging from 12.00 to 27.40 (on the z scale) showed a statistically significant moderating effect on life satisfaction – Table 5.

**Table 5:** Moderator values defining Johnson–Neyman significance region for the severity of menopausal symptoms/self-efficacy interaction on satisfaction with life.

GSES	Effect	SE	*T*	*P*	LLCI	ULCI
12.00	−1.82	0.63	−2.87	0.00	−3.06	−0.57
13.40	−1.69	0.59	−2.89	0.00	−2.84	−0.54
14.80	−1.57	0.54	−2.90	0.00	−2.63	−0.51
16.20	−1.44	0.49	−2.92	0.00	−2.41	−0.47
17.60	−1.32	0.45	−2.93	0.00	−2.20	−0.43
19.00	−1.19	0.40	−2.94	0.00	−1.99	−0.40
20.40	−1.07	0.36	−2.95	0.00	−1.78	−0.36
21.80	−0.94	0.32	−2.95	0.00	−1.57	−0.32
23.20	−0.82	0.28	−2.93	0.00	−1.36	−0.27
24.60	−0.69	0.24	−2.88	0.00	−1.17	−0.22
26.00	−0.57	0.21	−2.74	0.01	−0.98	−0.16
27.40	−0.44	0.18	−2.46	0.01	−0.80	−0.09
28.77	−0.32	0.16	−1.96	0.05	−0.64	0.00
28.80	−0.32	0.16	−1.95	0.05	−0.64	0.00
30.20	−0.19	0.16	−1.21	0.23	−0.51	0.12
31.60	−0.07	0.17	−0.40	0.69	−0.40	0.26
33.00	0.06	0.19	0.30	0.77	−0.32	0.43
34.40	0.18	0.22	0.82	0.41	−0.25	0.62
35.80	0.31	0.26	1.19	0.23	−0.20	0.81
37.20	0.43	0.30	1.46	0.15	−0.15	1.01
38.60	0.56	0.34	1.65	0.10	−0.11	1.22
40.00	0.68	0.38	1.79	0.07	−0.07	1.43

CI, confidence interval; LL, lower limit; UL, upper limit.

## 4 Discussion

In the study, it was examined whether self-efficacy acts as a moderator between the severity of menopausal symptoms and life satisfaction. The analysis of the relationship between generalized self-efficacy, satisfaction with life, and menopausal symptoms underscores the importance of considering both physiological and psychological aspects of women’s health during this period of life. Generalized self-efficacy refers to a person’s belief in his or her ability to perform tasks and cope with challenges in various difficult life situations (8, 21). Menopause symptoms are physiological and psychological changes experienced by women also in the postmenopausal stage of their lives (4). Our study results show that women with low levels of self-efficacy rate their quality of life worse. In the study group the average GSES score was 29.44 and was within the upper limits of the average reference values. It should be emphasized that people with high levels of generalized self-efficacy may approach menopause with a positive attitude, perceiving it as a natural stage of life. A woman’s belief in her ability to cope with menopausal symptoms can significantly affect her perception of this period (15). In turn, women with low levels of self-efficacy may feel overwhelmed by menopausal symptoms, viewing them as insurmountable obstacles that may negatively influence their quality of life (29).

The study results also showed a negative relationship between the vasomotor, psychosocial, and sexual domains of respondents’ quality of life and their sense of self-efficacy. Low levels of women’s self-efficacy may cause them to perceive the vasomotor or psychosocial symptoms of menopause, such as hot flashes, sweating, nervousness, mood swings, and poor memory, as particularly bothersome. These symptoms can cause women to feel tired and less able to effectively cope with everyday tasks. This, in turn, can undermine women’s confidence in their ability to function optimally and affect relationships with others leading to a decline in their quality of life (29). However, women with high levels of generalized self-efficacy may use more effective strategies to cope with menopausal symptoms. They may seek information and support, use complementary therapies to relieve menopausal symptoms, engage in a healthy lifestyle, and have a positive attitude. The study by Kafaei-Atrian et al. (29) found that increasing menopausal women’s self-efficacy for self-care can improve their health and thus quality of life. Vanden Noven et al. (30) discovered that the majority of perimenopausal and postmenopausal women take therapies to treat menopause-associated symptoms. Furthermore, this study found that exercise and mind–body therapies were the most commonly used to address sleep disorders, sadness, and anxiety. Research found that the improvement in self-care led to better adaptation to symptoms and enhanced quality of life (31, 32).

The general self-efficacy level indicates the individual’s resources, which support the individual in dealing with challenges in a variety of life domains. It allows the prediction of intentions in a variety of life domains, including health habits. Midlife women’s education, good attitudes, and management of menopause improve their quality of life during and after menopause. It has been shown that women with more negative attitudes toward menopause in general report more symptoms during perimenopause (33, 34). Interventions aimed at strengthening self-efficacy and positive attitude could lead to a lower symptom burden in menopausal women. Moreover, recognizing and comprehending individual characteristics that influence the burden of symptoms, such as self-efficacy, and attitude can make a substantial contribution to understanding the subjective experience of menopause and defining specific therapies (29). All these actions will aim to mitigate the impact of menopausal changes on women’s overall well-being (15). Also, as Yazdkhasti et al. (35), Parsa et al. (36) and Bahri et al. (37) emphasize in their study, self-care counseling for postmenopausal women improves their quality of life, and lack of information is a major challenge in dealing with problems resulting from menopause.

Analysis of the relationship between self-efficacy and the impact on the sexual domain shows that self-efficacy acts as a moderator affecting perceptions of change in this aspect of life. Whether a woman is married or has sexual relations while being widowed, divorced or a woman who is not in a relationship, the sexual domain affects a woman’s life and self-perception. Our study’s findings indicate that women who have lower levels of self-efficacy find sexually related symptoms to be more problematic. Similarly, Hsu et al. (38) proved that women with high levels of self-efficacy can approach sexual problems with greater confidence and a proactive attitude They may be more willing to seek information on how to cope with problems, lead a healthy lifestyle, but also talk to their partners about their needs and concerns, which, as Riazi et al. (39) point out, can affect the higher quality of their sexual life. The proactive approach may make it easier to maintain a satisfying sexual relationship despite the challenges posed by menopause. Likewise, Nazarpour et al. (40) show in their study that women with low self-efficacy may have more difficulty affirming their sexual needs, which can lead to reduced sexual activity, strained relationships with partners, lower satisfaction, and even the development of sexual dysfunctions.

In the study, the analysis of satisfaction with life among menopausal women in Poland showed values indicating average satisfaction with life, a similar level was presented in the Pałucka et al. (41) study conducted among Polish women while the study by Süss et al. (21) conducted as part of a research project in Switzerland indicated a high level of satisfaction with life in this group of respondents. The difference in satisfaction with life between menopausal women in Poland and Switzerland may have been caused by population differences. Furthermore, Switzerland, as a very highly developed country in terms of research and medical care, may probably offer better support for this group of women causing their life satisfaction to be at a higher level. The greater economic stability of Swiss women may also influence their higher overall life satisfaction.

Analysis of individual subscales of quality of life showed that female respondents rated quality of life as lowest in the psychosocial domain, and highest in the vasomotor domain. Thus, more than vasomotor symptoms such as hot flashes or night sweats, the lowering of quality of life among menopausal women was influenced by symptoms related to psychosocial functioning, including a need to be alone, a lack of patience for other people, anxiety, and irritability, or impressions of achieving less compared to the past. However, it should be noted that the relation between psychosocial functioning and quality of life assessment can be complex. It is important to take into account potential mistakes that may arise from participants’ perceptions when assessing this relationship. Quality of life assessment is a subjective evaluation that can be influenced by life goals and values, cultural norms, availability of social support, and ability to cope with stress, among other factors. Also, state of health, socioeconomic status, past life experiences, can affect the assessment of psychosocial functioning (16, 35, 42–44).

As the results of the study also show, women who experience more frequent mood swings, and depression, among other things, reported lower levels of life satisfaction. In practice, it means that psychosocial aspects might have a significant impact on menopausal women’s experience of life satisfaction, which may be important for developing interventions to improve their quality of life during this period (21). Czarnecka-Iwańczuk et al. (45) showed that women with more severe menopause symptoms in the psychological domain have lower self-esteem. Moreover, women experiencing more frequent and intensive somatic menopausal symptoms have lower satisfaction with life.

Better assessment of vasomotor quality of life may be influenced by the fact, that menopausal symptoms can occur in varying degrees of intensity in women and may decrease or subside over time. Moreover, these symptoms can be relieved by hormonal therapy, herbal supplements, lifestyle changes, etc. It should be emphasized that hormone therapy is the most effective treatment for postmenopausal symptoms. Non-hormonal pharmacologic, complementary, alternative, and behavioral treatments are available with varying efficacy and safety (46). These methods can be helpful in reducing the severity of symptoms and thus affecting a better quality of life in this domain (4, 47, 48). However, the issue of the impact of hormone replacement therapy or antidepressant treatment on women’s quality of life has not been investigated, due to the limitations of the study’s assumptions and objectives. These factors will certainly be taken into account in determining the course of our next study. Barati et al. (42) had a different opinion, they showed that the most influential in reducing the quality of life were vasomotor symptoms. Whereas in a study conducted in women living in rural areas, Kang et al. (49) indicated the highest quality of life in psychosocial domains and the lowest pointed to physical symptoms. The difference in the level of quality of life across scales may be due to the place of residence. In our study, the majority of women 68.6% were city residents, and they may have had better access to medical care offering methods to relieve menopause symptoms in the vasomotor domain. In contrast, the high level of quality of life in the psychosocial domain in women living in rural areas, as highlighted in a study by Kang et al. (49), may be due to the nature of those living in rural areas. They are helpfulness, do not feel uncomfortable about asking for help, and appreciate the help received from someone. This makes them more satisfied in their interpersonal relationships compared to female urban residents, which may also translate into the overall perceived quality of life in this domain.

According to the literature, the functioning and quality of life of menopausal women require the use of multifaceted methods or strategies for coping with the symptoms and consequences of this period. These include, in addition to the pharmacotherapy, physical and mental health education, psychological and social support (35, 50). One of the elements of a health-promoting lifestyle is physical activity, which is one of the methods of relieving the menopausal symptoms (51–53). Regular physical activity (resistance training, aerobic training, stretching, relaxation) has a positive impact on well-being, prevents osteoporosis, reduces the risk of chronic diseases or allows women to maintain a healthy body weight. Moreover, regular physical activity combined with limiting coffee and alcohol and not smoking cigarettes helps reduce hot flashes and night sweats (51). It is important to take into account the results of the Dabrowska-Galas and Dabrowska (51) study, which showed that the level of physical activity is linked to self-esteem. The middle-aged women surveyed (between the ages of 45 and 60), were characterized by both greater physical activity and higher self-esteem. Their findings indicate the need to integrate physical activity (PA) into the lives of middle-aged women to improve their self-esteem and mental health (51).

Diet is another extremely important component of the lifestyle of menopausal/peri-menopausal women, as it affects the symptoms associated with this period, as well as the quality of life (54–56). A plant-based diet and restriction of oils can significantly reduce the incidence of moderate to severe postmenopausal hot flashes and their associated symptoms. In addition, it is accompanied by weight loss and improvements in the physical, psychosocial and sexual domains. Products containing phytoestrogens can help relieve hot flashes and night sweats by imitating the effects of estrogen in the body (55, 56). Increased intake of calcium and vitamin D prevents loss of bone mass and risk of osteoporosis. Consumption of foods rich in omega-3 fatty acids support brain health and improve mood, by helping to relieve the mental symptoms of menopause, such as mood swings and depression (57). Proper hydration by drinking lots of fluids can help combat dry skin, vaginal dryness and hot flashes (58). In turn, avoiding caffeine, alcohol and spicy foods, for example, reduces the severity of hot flashes (59).

Analysis of the relationship between life satisfaction and the severity of menopausal symptoms determining women’s quality of life showed that reduced life satisfaction in menopausal women was significantly associated with a decrease in overall quality of life, as well as quality of life in the psychosocial and sexual scales. A lower level of quality of life associated with more strongly felt menopausal discomforts among the women surveyed influenced a lower sense of life satisfaction. Bien et al. (60) in their study, on the quality and satisfaction with life of women who are childless by choice, showed that there was a positive correlation between life satisfaction and overall quality of life, overall perception of health, and all domains of quality of life - physical, psychological, social relationships, environmental. Moreover, higher life satisfaction among respondents was correlated with higher quality of life scores and better-perceived health (60).

Sexual activity is another important element of quality and satisfaction with women’s lives. Most women between the ages of 40 and 60 are sexually active. However, the peri-menopausal changes occurring at this time can also affect this sphere of life. Women who present positive attitudes toward sexual life during the peri-menopausal period are usually characterized by greater self-confidence and greater self-acceptance. Higher satisfaction with the sexual domain of life also results in better relations with the partner (41, 43, 61). Thus, it can be assumed that a higher quality of life in the sexual domain determines higher overall life satisfaction. The results of our study presenting the influence of stronger symptoms on the sexual scale on lower life satisfaction also lead to the same conclusion. These results are consistent with those of Ornat et al. (62) who showed that in menopausal women, the level of life satisfaction is positively related to better sexual functioning.

The first hypothesis was confirmed, which assumed that a decrease in life satisfaction (both in terms of experienced psychophysical well-being and the ability to achieve life goals) in menopausal women significantly co-occurs with an increase in the severity of psychophysical symptoms of menopause. There are several implications resulting from the relationship between the severity of menopausal symptoms and life satisfaction. Firstly, women who experience a lot of loss owing to severity of menopausal symptoms are more likely to experience further losses, and consequently to experience a rapid or successive reduction in life satisfaction (15). Loss is understood as a negative consequence or loss associated with menopausal symptoms that affect various aspects of a woman’s life. Loss can include a variety of meanings and aspects, such as loss or threats of valued goals and values related to a woman’s role, fertility, femininity, weakening of family bonds, social support, deterioration of health, physical fitness, and emotional well-being (63, 64). Secondly, the risk of reduced quality of life for women will increase when the occurrence of menopausal symptoms generates severe stress - due to overload, anxieties, conflicts, and difficulties in achieving goals (16). Thirdly, a high risk of reduced life satisfaction for women is the long-term experience of menopausal symptoms, because, with each successive loss, they will have a smaller pool of resources to use to defend themselves against stress. The risk of reduced quality of life may also stem from the feedback loop between menopausal symptoms and experienced stress. The high intensity of menopausal symptoms causes severe stress, and high levels of stress lead to the strengthening of existing menopausal symptoms (16). However, the decrease in the quality of life during the climacteric period may also be enhanced by the occurrence of stressors of everyday life (2, 15, 65, 66). The result is consistent with the results obtained in other studies on the relationship between the presence of menopausal symptoms and the level of quality of life and satisfaction with life in menopausal women (2, 14, 66, 67).

Hormonal changes that accompany the menopausal transition can affect the mood of women during this period (68–70). Kuck and Hogervorst’s (71) study found that women in early perimenopausal period experienced the highest levels of stress, as well as more feelings of depression and anxiety, and their quality of life in the psychosocial domain was the worst. Postmenopausal women reported similar experiences to those before menopause. On the other hand, age explained the associations between menopause, stress, and anxiety, but not between depression and different stages of menopause (71). A study by Süss et al. (21) that aimed to uncover psychosocial factors promoting resilience among perimenopausal women should be highlighted here. They found that optimism, emotional stability, emotion regulation, self-compassion and self-esteem could be attributed to a single factor associated with resilience. Furthermore, according to their study, women with higher resilience seem to have better well-being and good mental health during this period. In addition, it is generally a time of heightened stress, where stressors occur and accumulate, which vary by time of occurrence, that is, acute stressors like watching disturbing news, everyday events/problems - living in a constant rush, stressful life events like the loss of a loved one, or chronic stressors in the form of performing long-term caregiving duties or financial problems. Therefore, it is also a time of experiencing stressful life events that affect the balance of the body. Because of the close interaction of the reproductive- and the stress axes, stress can act as a trigger or perpetuator of disorders such as depression or insomnia (68).

In our study, high self-efficacy is positively associated with satisfaction with life, which states that higher life satisfaction is shown by women who cope better with the difficulties encountered in life. One of the mechanisms through which self-efficacy affects life satisfaction is its effect on coping strategies. Menopausal women who believe in their ability to successfully cope with life’s challenges and difficulties will be more likely to solve problems and overcome potential difficulties. Having this approach can lead to a higher sense of control over one’s life and higher self-confidence, which will be reflected in higher levels of life satisfaction (21, 72). Similar relationships can be found in studies by other authors assessing the impact of the level of coping with difficult situations on particular psychological aspects of a person’s life, such as motivation or life satisfaction (72, 73). They note, however, that it is necessary to be very careful before implementing these results for the general population. As the level of this relationship can also be influenced by several individual factors such as personality traits and environmental conditions, it cannot be determined at a constant level and applied to everyone equally (72, 74).

The results of the conducted empirical analyses also confirmed the second of the hypotheses, which assumed that the sense of self-efficacy in women during menopause plays a moderating role in the relationship between the intensity of menopausal symptoms and the level of life satisfaction. The moderating function of self-efficacy is that the increase in menopausal symptoms: at low intensity of self-efficacy lowers life satisfaction, at medium and high intensity of self-efficacy does not reduce life satisfaction. The obtained results are confirmed by data from the literature, which indicate the existence of factors inhibiting the reduction of life satisfaction in women during menopause. These could include living in a city, having a better socioeconomic status, a career, being in a stable relationship, having fewer psychological symptoms, or getting more sleep (14–17).

The individuals with higher levels of self-efficacy experience a reduced impact of menopausal symptoms on life satisfaction. Self-efficacy is a resource for both building long-term resilience to stress, tough situations, as well as a resilience resource that directly contributes to constructive coping with stress generated by menopausal symptoms (24, 25, 75). It is necessary to keep in mind that the relationship between generalized self-efficacy and satisfaction with life and menopausal symptoms is complex and multi-dimensional. While self-efficacy can affect how women perceive and cope with menopause, the severity and nature of menopausal symptoms can also affect their sense of efficacy. Interventions to support menopausal women should therefore address both their physiological symptoms, self-care during this period, and psychological resources, particularly self-efficacy (29, 71).

The interventions aimed at boosting self-efficacy, such as educational activities, cognitive-behavioral therapy, and mindfulness-based approaches, may be beneficial for women experiencing negative menopausal symptoms (76, 77). They can challenge negative beliefs about menopause and foster a more positive approach to the period. Activities that strengthen women’s confidence in their ability to successfully cope with difficulties and challenges can help improve their satisfaction as well as their overall quality of life during this unique period of life (44, 78). According to a study by Shobeiri et al. (44) these activities can improve women’s quality of life in the vasomotor, psychosocial, sexual, and physical dimensions (in dimension).

The weakness of our research is that it was in a correlation scheme, as a consequence of which it is not possible to draw cause-and-effect conclusions based on the obtained results. The presented results are exploratory. Furthermore, we have not analyzed the correlation between the use of appropriate therapy, type of menopause, levels of generalized self-efficacy, and severity of symptoms. We are also aware that, as the literature shows, self-efficacy and satisfaction with life are influenced by factors we did not analyze in this article, such as career, psychological disorders and amount of sleep, and women’s individual experiences of menopause are very diverse (14–17). Moreover, the severity of menopause-related symptoms and women’s perceptions of them also interact with other psychological, cultural, and socioeconomic factors. The study was conducted in only one region in Poland, so the generalization of our findings to other areas and ethnicities should be made with caution.

## 5 Conclusion

Menopausal women have an average level of self-efficacy and an average level of life satisfaction. There is a relationship between the intensification of symptoms of menopause and the reduction of life satisfaction in menopausal women. Self-efficacy moderates the relationship between the severity of menopausal symptoms and life satisfaction in menopausal women. The moderating function of self-efficacy is that at its low level, an increase in the severity of menopausal symptoms reduces life satisfaction, while at medium and high levels, an increase in the severity of menopausal symptoms does not reduce life satisfaction.

## Data availability statement

The original contributions presented in the study are included in the article/supplementary material, further inquiries can be directed to the corresponding author/s.

## Author contributions

AB: Supervision, Project administration, Conceptualization, Writing – original draft. IN: Writing – review & editing, Writing – original draft, Methodology, Conceptualization. MK-P: Writing – review & editing, Writing – original draft. ER: Writing – original draft, Investigation. MZ: Writing – review & editing, Visualization. BP: Writing – review & editing. KJ: Writing – review & editing, Methodology, Formal analysis.
